# The Scale of Faith Based Organization Participation in Health Service Delivery in Developing Countries: Systemic Review and Meta-Analysis

**DOI:** 10.1371/journal.pone.0048457

**Published:** 2012-11-12

**Authors:** Rose Calnin Kagawa, Andrew Anglemyer, Dominic Montagu

**Affiliations:** 1 Global Health Group, University of California San Francisco, San Francisco, California, United States of America; 2 Global Health Sciences, University of California San Francisco, San Francisco, California, United States of America; UNAIDS, Switzerland

## Abstract

The extent of faith-based organizations' participation within the overall health systems of developing countries is unclear. Recent reports state that faith-based organizations play a substantial role in providing healthcare in developing countries, cited in some publications as up to 70% of all healthcare services. The data behind these numbers are sometimes difficult to pinpoint and seem at odds to national and regional survey data. In an effort to quantify the contribution of faith-based organizations to healthcare delivery in low- and middle-income countries, we undertook a systematic review of the literature and conducted a new analysis of relevant Demographic and Health Survey data from 47 countries. Our findings demonstrate that the magnitude of healthcare provided by faith-based organizations may be lower than previously estimated. Understanding the scale of FBO-provided medical care is important for health sector planning, and more accurate and complete estimates are needed.

## Introduction

Faith-based organizations (FBOs) are considered an important partner in health-systems strengthening and assuring equity of access to healthcare in developing countries. For decades churches played an important role in low- and middle-income country health services. During the eighteenth and nineteenth centuries, mission societies began providing medical aid under colonial governments in Asia, Africa, and Latin America. These historical roots remain evident in the continued presence and stature of FBOs in developing countries. As health systems evolved and social services of all kinds became a core component of national social system structures, the relative importance of FBOs within the broader structure of health services of developing countries has become less certain.

The contribution of private for-profit providers has grown in the past decades and represents the majority of care in most developing countries [1]. Less clear is the scale of FBOs within the overall health systems of developing countries today. Recent reports have stated that faith-based organizations play a substantial role in care in developing countries [2]–[4], cited in some publications as up to 70% of all services [2]. The data behind these numbers are sometimes difficult to pinpoint and seem at odds to national and regional survey data [5], [6]. Many studies have noted the lack of robust data on faith-based health care services and the need for more methodologically sound estimates to inform policy [7]–[9]. At least one of these studies has undertaken the exercise of describing the scale of FBO-provided care, though in the authors' own words, the review is a “profiling exercise” rather than a “comprehensive, detailed survey” [3].

In order to provide a clearer picture of the role of FBOs in health systems strengthening, we undertook a systematic review of existing literature and paired the results with a new meta-analysis of Demographic and Health Survey (DHS) data [10] from 47 countries. To the best of our knowledge, the resulting data, from 59 sources and covering 48 countries, provides the first comprehensive assessment of the current importance of FBO-provided medical care in developing countries to attach only verifiable data to the estimates of FBO-provided care.

## Methods

### Searching

Between September 26 and December 1, 2011 we conducted a systematic review of the literature from the past 11 years. We searched databases including PubMed and Google Scholar, the USAID-supported Health Systems 20/20 website, the World Health Organization website, and the World Bank website. We also used a general Google search to find grey literature on the subject. Finally, we included sources identified by personal contacts and sources identified in the bibliographies of papers from our original search.

Our PubMed search included the following Mesh terms: (“Hospitals, Religious”[Mesh] OR “Missions and Missionaries”[Mesh] OR “Medical Missions, Official”[Mesh]) AND (“Ownership”[Mesh] OR “Health Services Research”[Mesh] OR “Regional Health Planning”[Mesh] OR “Hospital, Private”[Mesh]).

In Google Scholar we searched article titles for the following: health AND “faith based” OR “faith-based” OR mission OR religious OR church OR “health system planning” OR “health sector planning” OR “health sector survey” OR “health system assessment” OR “health services research”.

### Selection

Our goal was to identify documents and government data that quantified the magnitude of faith-based care in developing countries. To meet our criteria, faith-based care could be enumerated as provision of healthcare services or contribution of clearly defined infrastructure to the national health system. For inclusion, we required that sources describe methodologies that would reduce bias in their estimates of the magnitude of faith-based care or infrastructure in each country. These methodologies could include extensive desk reviews combined with key informant interviews, facility mapping, or questionnaires. We limited our search to include papers published after January 1, 2000 in order to include the most up-to-date estimates.

Publications were excluded if they did not provide national-level data and specifically quantify the contribution of faith-based organizations.

### Validity Assessment

Only papers that specifically described their sources and data collection methodology were included. Sources providing unreliable citations for their data were removed.

### Data Abstraction

Using available data from Demographic and Health Surveys, we synthesized the data across countries in an attempt to quantify the true estimate of the magnitude of healthcare provision by FBOs. In order to provide the most conservative estimates, we developed regional aggregates using the upper bound of the 95% confidence interval around the point estimate calculated from the DHS data for each country. We estimated the proportion of outpatient healthcare provided by FBOs using data that describe treatment sought for diarrhea or cough as a proxy for all outpatient health care. We estimated the proportion of inpatient healthcare provided by FBOs using data that describe deliveries as a proxy for all inpatient healthcare. We performed subgroup analyses to explore the impact regional differences may have on summary estimates. All meta-analyses were performed in R 2.14.0 and modeled using a DerSimonian-Laird random-effects model [11], weighting country estimates by their respective population sizes as determined by the World Bank [12].

## Results

### Flow of Included Studies

**Figure 1:**
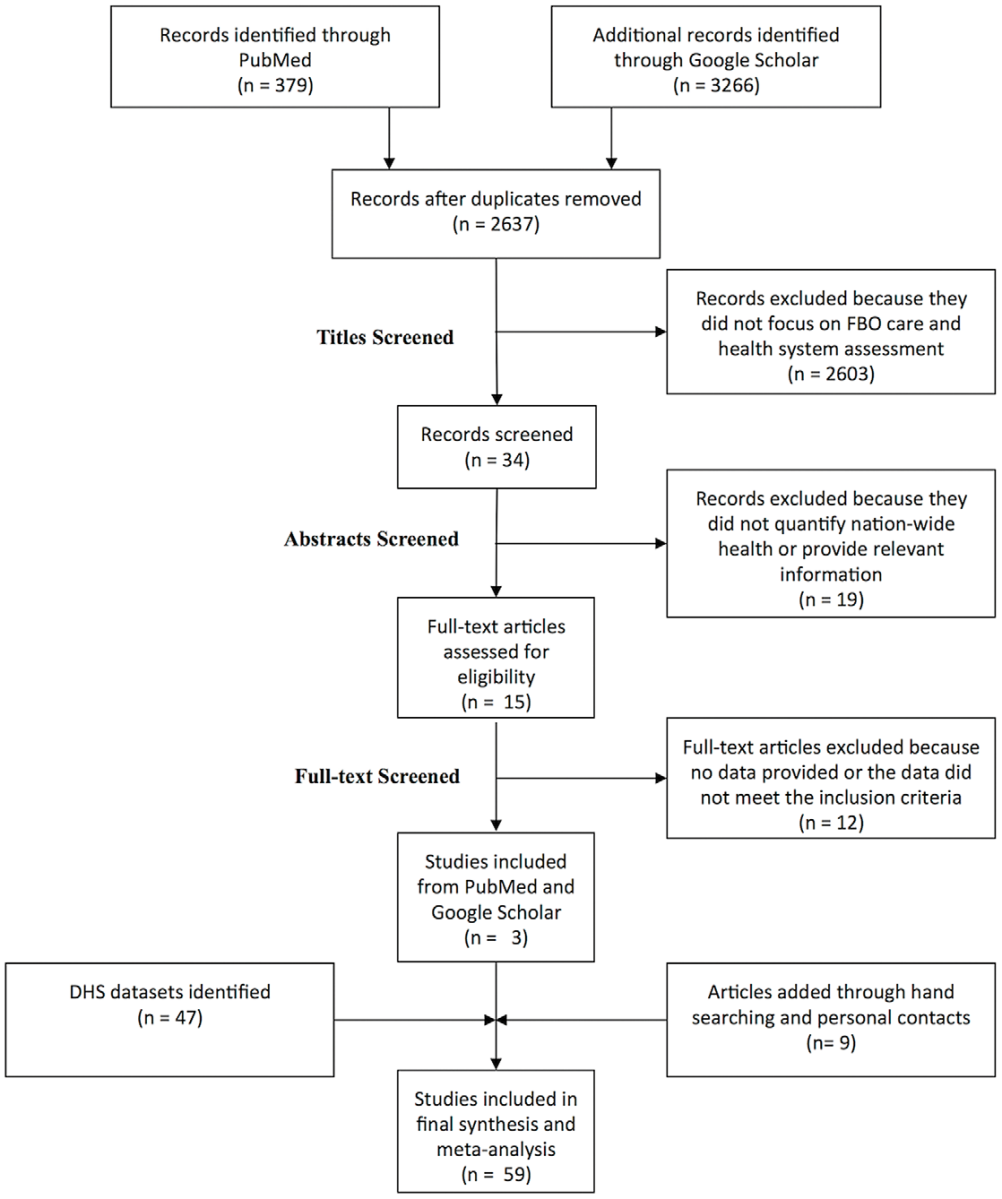
Study Selection PRISMA Diagram [13].

The initial literature search yielded 3,645 sources. After removing duplicates and ineligible studies based on a review of the titles, abstracts and full texts, we had three studies remaining. Additional hand searching and the inclusion of eligible sources identified through personal contacts added nine sources to the list (see Figure 1).

For the quantitative analysis, we identified 47 sets of DHS data through USAID resources. Other sources of country-level data (e.g. World Health Surveys (WHS), National Health Accounts (NHA) and Multiple Indicator Cluster Surveys (MICS)) were identified through our search and considered for inclusion in the quantitative analysis. However, these sources of data were ultimately not included due to a lack of analyzable data. Faith-based organizations represent a subset of private healthcare and the contribution of FBOs to total private healthcare provision varies across countries. None of these datasets specifically identified FBO-owned facilities or FBO-provided care, tending instead to lump FBOs with other, generally private sources of care. Therefore, we did not include these data in our analysis.

Our final review includes 59 sources (including 47 DHS datasets) that quantify the contribution of faith-based organizations to national healthcare services in a total of 48 countries.

### Study Characteristics

The studies included in the final qualitative review can be grouped into two categories: established surveys conducted by multilateral organizations including the Health Systems Assessment Approach (HSA), Service Provision Assessment Surveys (SPA), and Service Availability Mapping (SAM); and grey literature and primary data including Ministry of Health records and a conference paper. The latter are less explicit in their methodology, though their estimates fall within the range of estimates provided by the former studies. See Appendix S1 for a description of the methodologies of identified data sources.

**Figure 2:**
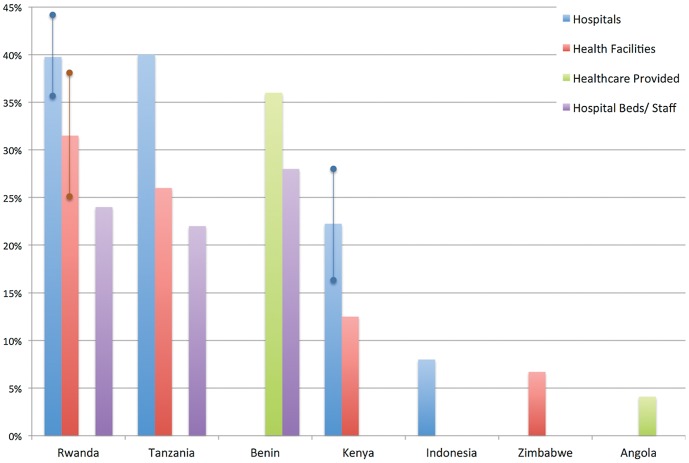
The Percentage of all Healthcare Services or Infrastructure in Low- and Middle-Income Countries that is Provided by Faith-Based Organizations. Ranges are shown where they were included in the original data. Source: Systematic Review [14]–[25].

We describe the proportion of healthcare provided by FBOs as it pertains to the percentage of total number of hospitals, the percentage of total number of health facilities, the percentage of total healthcare provided, the percentage of total hospital beds, or the percentage of total staff of the national healthcare system (Table 1 and Figure 2).

**Table 1:** Summary of Reported Proportion of Healthcare Provided by Faith-Based Organizations (2000–2011).

Hospitals	Health Facilities	Hospital Beds/Staff	Healthcare Provided
8% Indonesia MoH 2011	**12.5%** Kenya NCAPD 2011 Luoma 2010	**28%** Benin Adeya 2007	**4.1%** Angola Connor 2010
16.5% Kenya NCAPD 2011 Luoma 2010	**25%** Rwanda NIS 2008	**24%** Rwanda Schneider 2000	* **36%** Benin Adeya 2007
28% Kenya Wamai 2004	**38%** Rwanda Schneider 2000	**22%** Tanzania MOHSW 2007	
44% Rwanda MoH 2003	**26%** Tanzania Todd 2009		
35.5% Rwanda Schneider 2000	**6.7%** Zimbabwe Osika 2010		
40% Tanzania Todd 2009			
Range **8–44%**	**6.7–38%**	**22–28%**	**4.1–36%**

*Percent contributed by FBOs, country, author, date published.*

* Percent of hospitalizations.

These data come from sources published since 2000 that quantify faith-based contributions specifically and use a verifiable methodology including Health Systems Assessment Approach, spatial mapping, Private Health Sector Assessment, Service Availability Mapping, and other surveys and desk reviews [14]–[25].

### Qualitative Data Synthesis

The 12 studies included in the literature review demonstrate that the national-level proportion of religious healthcare services and infrastructure varies widely across countries and across units of measurement. The discrepancy in estimates points to the difficulty of quantifying the contributions of FBOs to national healthcare delivery. The majority of included studies focus on Africa (11 of 12), while Indonesia is the only non-African country included (see Table 1 and Figure 2). Across all indicators, the magnitude of FBO contributions ranges from 4.1 percent in Angola to 44 percent in Rwanda. Different units of measurement between and within countries add greatly to this heterogeneity.

Depending on the unit of measurement, the proportion of religious medical efforts varies widely. All the studies included quantify religious contributions by measuring infrastructure in a given country such as hospitals, health facilities, hospital beds, or health staff, or by measuring the proportion of national healthcare provided by FBOs. Measurements using hospitals as the unit of analysis tend to produce a higher estimate than other measurements. In Kenya, Rwanda, and Tanzania, Eastern African countries from which we have multiple measurements, the proportion of FBO-owned hospitals is consistently larger than the proportion of services provided or infrastructure-owned by FBOs. In Kenya, the percentage of FBO-owned hospitals is reported as 16.5–28 percent while the percentage of FBO-owned health facilities is 12.5 percent. In Rwanda, FBOs own 35.5–44 percent of hospitals, 25–38 percent of health centers or facilities, and 24 percent of hospital beds. Similarly, in Tanzania FBOs own or manage 40 percent of hospitals, 26 percent of health facilities, and 22 percent of health staff. In contrast, Connor et al. attribute only 4.1 percent of all healthcare provided in Angola to FBOs [14]. The variation in these estimates could be the result of historical mission emphasis on hospital-based care and limited FBO investment in lower-level facilities. Whether or not that is the case, the results highlight the potential discrepancy in measures of supply of health infrastructure versus measures of use of health services.

Our systematic review yielded estimates quantifying the supply of health infrastructure, hospital beds, and health staff as well as estimates of the proportion of healthcare services provided by faith-based organizations. The measures of healthcare provided are more similar to the DHS measures of service utilization. Our review includes two utilization measures: FBOs in Angola provide 4.1 percent of healthcare, and FBOs in Benin accommodate 36 percent of hospitalizations. The estimate for Angola is considerably lower than other measures in our review and more similar to the estimates produced through the analysis of DHS data. We do not know whether this is because the role of FBOs in Angola is limited or because the measure of utilization yields a systematically lower result. Benin however gives an unexpectedly high proportion of hospitalizations compared to the proportion of bed capacity owned by FBOs. Adeya et al. attribute this imbalance to higher occupancy rates at FBOs and considerable unused bed capacity throughout the system [15]. Though higher than expected, the estimate of hospitalizations is still lower than the estimates of the proportions of hospitals and health facilities owned and managed by FBOs in Benin. This follows the pattern of measures of utilization producing lower estimates than measures of supply [14]–[25].

The Christian Health Association (CHA) of Africa has published similar data on the contribution of Christian organizations to national healthcare systems in Africa. Christian Health Associations serve as the umbrella organization for Christian-related health programs and services in many countries in Africa. Estimates from CHAs report that Christian health networks contribute between 30 and 55 percent of health facilities in their respective countries. Though a graph of CHA data describing the proportion of Christian health facilities by country appears in a number of publications [26]–[28], these data were not included in this review because they were presented without sources or clear methodologies. Published estimates of the contributions of FBOs to national healthcare identified in our literature review are consistently lower than the CHA data.

### Quantitative Data Synthesis

DHS datasets were identified in our search and used for the present quantitative analysis. Other sources of country-level data were considered for this analysis, including datasets from WHO (WHS and NHA data) and UNICEF (MICS), but they did not provide adequate quantitative data for inclusion in the meta-analysis.

### Outpatient Care

**Figure 3:**
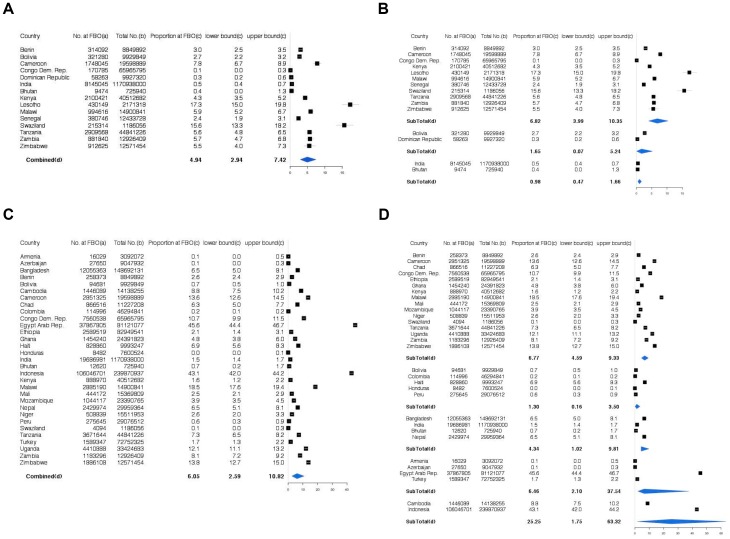
Forest Plots of Outpatient and Delivery Care Provision across All Countries and Regions Reporting Any FBO-Provided Healthcare in DHS Data. Panel A: Outpatient Care Provision Across All Countries; Panel B: Outpatient Care Provision Across All Countries Stratified by Region; Panel C: Delivery Care Provision Across All Countries; Panel D: Delivery Care Provision Across All Countries Stratified by Region. a) Number at FBO was back-calculated from the upper bound of the confidence interval surrounding the proportion estimate from the DHS reported data. b) Population sizes are World Bank's most recent (2010) population size estimates. c) Estimated from DHS reported data. d) Sub-totals are pooled estimates using random effects models using the upper bound for the country-specific estimates.

For outpatient care, as defined by treatment sought for pediatric diarrhea or cough, we calculated the summary estimates for countries in which at least one individual noted having sought healthcare at a FBO-affiliated facility in DHS surveys (Figure 3, Panel A). Across these facilities, the estimated weighted summary proportion of outpatient healthcare provided by FBOs was 4.9% (95% CI 2.9–7.4%). The range of reported country estimates from these DHS datasets was from 0.1% (Congo DR) to 17.3% (Lesotho). We also calculated estimates of outpatient healthcare provision by FBOs among all countries, including countries from which DHS surveys suggested no patients sought FBO health facility care. Across all countries, the estimated proportion was 0.5% (95% CI 0.2–0.8%).

#### Regional Subgroups

To estimate the impact regional differences contribute to the heterogeneity of the summary estimates, we performed subgroup analyses by region (see Figure 3, Panel B). The five regions include: Sub-Saharan Africa, Latin America, South-East Asia, South Asia, and a combined region of Asia/North Africa/Europe (see Appendix S2 for country break-downs). The estimates in Figure 3, Panel B only include countries in which at least one individual in the DHS surveys noted having sought outpatient care at a religious affiliated facility. The highest proportion of reported FBO healthcare provision was in Sub-Saharan Africa (6.8%; 95% CI 4.0–10.4%). In Latin America the estimate was 1.7% (95% CI 0.1–5.2%) and in South-East Asia 1.0% (95% CI 0.5–1.7%). When considering all countries, including countries with no reported FBO care provision, the estimated proportions were 1.2% in Sub-Saharan Africa (95% CI 0.4–2.3%), 0.2% in Latin America (95% CI 0.0–0.9%), and 0.2% in South Asia (95% CI 0.0–0.8%). No countries in South Asia or in the Asia/North Africa/Europe regions had reportable data of FBO care provision in the DHS datasets.

### Delivery (Inpatient Care)

For inpatient care, as defined by deliveries, we calculated the summary estimates for countries in which at least one person reported having sought healthcare in a religious facility in DHS surveys (see Figure 3, Panel C). Across these countries, the estimated proportion of delivery care provided by FBOs was 6.1% (95% CI 2.6–10.8%), and the range of reported country estimates was from 0.0% (Honduras) to 45.6% (Egypt). Additionally, we calculated estimates of deliveries at FBOs among all countries, including countries from which DHS surveys suggested no patients sought care at a faith-based health facility. Across all studies, the estimated proportion was 2.5% (95% CI 0.8–4.9%).

#### Regional Subgroups

To estimate the impact regional differences contribute to the heterogeneity of the summary estimates, we performed subgroup analyses by region (see Appendix S2 for country descriptions). Estimates in Figure 3, Panel D are among countries in which at least one individual reported having delivered at an FBO in DHS surveys. The highest proportion of reported FBO care for deliveries was among countries in South Asia (25.3%; 95% 1.8–63.3%), followed by Sub-Saharan Africa (6.8%; 95% CI 4.6–9.3%), and the combined region Asia/North Africa/Europe (6.5%; 95% CI 2.1–37.5%). In Latin America the estimate was 1.3% (95% CI 0.2–3.5%), and in South-East Asia 4.3% (95% CI 1.0–9.8%). When considering all countries, including countries with no reported FBO care provision for deliveries, the estimated proportions were 11.8% in South-East Asia (95% CI 3.7–60.9%), 1.64% in Asia/North Africa/Europe (95% CI 0.2–14.2%), 2.41% in Sub-Sahara Africa (95% CI 1.0–4.5%), 2.8% in South Asia (95% CI 0.5–6.9%), and 0.9% in Latin America (95% CI 0.0–2.6%).

## Discussion

Faith-based organizations provide essential health infrastructure and healthcare in many countries in Africa and other regions of the world. However, the estimates of religious medical contributions vary widely across studies, units of measurement, and geographic regions. Our knowledge of the magnitude of faith-based contributions is limited and imprecise, making it difficult to define the role of faith organizations in health sectors globally. To the best of our knowledge, the studies reviewed here constitute the most accurate available, having used robust and verifiable assessment methodologies. However they remain imprecise, and their role in estimating the market share of FBO-provided care is more exploratory than exact.

Measuring the magnitude of the contribution of FBOs to national healthcare is further complicated by the prevalence of repeated dubious estimates. For example, the *Nigeria Health System Assessment* published that faith-based organizations in Nigeria provide 60% of the country's healthcare [29]. The report cites Marc Larbi and the team at the University of Birmingham who wrote a study on non-state providers of basic services. Upon further review, we found that Larbi et al. cite a personal communication with PATH in Enego as the source for the Nigerian estimate rather than a systematic study of healthcare services [30]. This reproduction of a shaky estimate is not unique, and it calls into question the estimates produced by other health sector assessments, and by non-critical synthesis reports.

Notably, this literature review includes only one non-African country - Indonesia. The paucity of data around the world points to a large gap in knowledge, yet we know religious hospitals play a role in many regions outside of Africa. In 1993, the World Development Report published that FBOs provide more than ten percent of clinical services in India, operate nearly half of hospitals in Haiti and own nearly 25 percent of health facilities in the three largest cities of Brazil [31]. Jordan, seven percent of the population receives care at mission hospitals and NGOs [32], and in Nepal, churches own 19 percent of the hospitals [33]. A 2010 review of FBOs in Latin America notes their importance in health and social service provision in the region, but does not offer quantified measures [34]. The DHS databases also demonstrate that religious healthcare providers are active in many countries not listed in our systematic review, but in none of these countries has a recent, rigorous study of the contribution of religious health assets been conducted.

Our literature review yielded published data that highlights differences between often-cited Christian Health Association data and published, peer-reviewed data. Published estimates identified in our literature review were consistently lower than the CHA data. These differences could be due to differing definitions of what constitutes a health facility or perhaps illustrate the difficulty in quantifying religious health assets. Patients may be unaware of the ownership of the facilities they visit, and end-users of data may have institutional biases that lead to over or under-estimation of the FBO sector. Despite these discrepancies among data sources, these differences once again point to imprecision in the estimates of the contributions of FBOs.

The meta-analysis yielded results that were presumably more conservative because we used the upper estimates from the confidence intervals for each respective country estimate of FBO-provided healthcare use. Though using the lower estimates from the confidence intervals for the meta-analysis would have yielded lower summary estimates for both inpatient and outpatient care, it was our intention with this analysis to allow for the possibility of under-reporting of healthcare source by DHS study participants. Additionally, we performed sensitivity meta-analyses whereby we only allowed countries in which at least one participant in the DHS surveys reported having received healthcare from an FBO to contribute to the summary statistics (see Figures 3a–3d). As expected, these sensitivity estimates were higher than the summary estimates across all countries (outpatient: 4.5% and 0.5%, respectively; inpatient: 4.9% and 1.8%, respectively).

Large differences in the estimates of the contributions of FBOs between the systematic review and the analysis of DHS data highlight the difference in measuring supply versus utilization of healthcare services. Measures of infrastructure, staff and bed capacity do not translate directly to utilization and may lead to overestimations of the proportion of healthcare provided by religious organizations.

### Limitations

The concept of the contributions of FBOs to national health services can vary from medical services provided (e.g. vaccinations) to palliative care (e.g. support groups for people living with HIV). Though we sought to only include data on medical services or infrastructure, some sources did not specify their definition of religious contribution. Additionally, the meta-analysis found substantial heterogeneity between countries for all estimates (p value <0.01); despite subgroup analyses by region the heterogeneity remained, suggesting that there are potentially other unknown sources of bias among study estimates. Furthermore, the populations at risk between the two main outcomes for meta-analyses are different. Specifically, for outpatient care we used the World Bank total population size estimates for each country as the population at risk, though the DHS data for these results were collected only for children under five (outpatient data). As such, these results may not reflect adult healthcare seeking behavior. In turn, though the country-specific proportions reported are unaffected, the weight given to each country for the meta-analysis could be artificially overestimated or underestimated relative to all other countries if its population distribution of age is different. Similarly, for the meta-analyses regarding deliveries, the proportion reported in the DHS data was among all institutional deliveries, while the population used for the meta-analysis was the total population. Again, as a result, the weight given for the meta-analysis to any given country could be artificially overestimated or underestimated relative to all other countries if its population distribution of gender is different. Additionally, the years for the collected DHS data range from 2003 to 2008; the most recent DHS data for each country were used for this analysis. Finally, the systematic review relied on one primary source of quantitative data, the DHS. While we made every effort to identify all potential sources of healthcare provision data on a country-level, we may have unintentionally missed some sources of data. The data presented in the systematic review, though methodologically rigorous, may itself be subject to a form of publication bias: to under or over reporting as a result of prioritization by religious groups or by donors or governments. The scale of FBO services in LMICs is often a politically charged issue, and it is possible that political context effects data availability.

Faith-based organizations are an important component of healthcare in many developing countries around the world, particularly in Africa. Our findings suggest that the importance in health delivery and infrastructure may be lower than has previously been estimated, but confirm the relevance of FBOs to health systems in many countries. More rigorous work is needed to clarify FBO activities, both in clinical care and beyond, and so allow health policy makers around the world to develop national and regional health plans that appropriately reflect the contributions and potential of FBOs within the overall health system. We are optimistic that the growing attention being paid to better measure FBO contributions to health systems will lead to improvements in performance tracking and funding allocations.

## Supporting Information

Appendix S1 **Methodology of the Sources.**
(DOCX)

Appendix S2 **DHS Regional Breakdown.**
(DOCX)
