# Correction: The Scale of Faith Based Organization Participation in Health Service Delivery in Developing Countries: Systemic Review and Meta-Analysis

**DOI:** 10.1371/annotation/1e80554b-4f8a-4381-97f1-46bf72cd07c9

**Published:** 2012-11-29

**Authors:** Rose Calnin Kagawa, Andrew Anglemyer, Dominic Montagu

The word "Systematic" is misspelled in the title. The correct title is: The Scale of Faith Based Organization Participation in Health Service Delivery in Developing Countries: Systematic Review and Meta-Analysis. The correct citation is: Kagawa RC, Anglemyer A, Montagu D (2012) The Scale of Faith Based Organization Participation in Health Service Delivery in Developing Countries: Systematic Review and Meta-Analysis. PLoS ONE 7(11): e48457. doi:10.1371/journal.pone.0048457 

