# Supplementary material for: The Scale of Faith Based Organization Participation in Health Service Delivery in Developing Countries: Systemic Review and Meta-Analysis
Source: PLoS One. 2012 Nov 12;7(11):e48457. doi: 10.1371/journal.pone.0048457 (PMC3495941; doi:10.1371/journal.pone.0048457)
Supplement: Appendix S1 — Methodology of the Sources. (DOCX) [file pone.0048457.s001.docx]

**Appendix S1: Methodology of the sources**

The Health Systems Assessment Approach (HAS) used in Angola, Kenya and Zimbabwe measures service delivery and health information systems among other topics. The HSA approach generally includes a combination of primary data collection, document review, key informant interviews, and health facility visits.

Service Provision Assessment (SPA) surveys were used in Rwanda and Kenya. The SPA survey is conducted among a sample of health facilities including hospitals, health centers and dispensaries. Health service provider and client interviews, facility audit questionnaires, exit interviews with clients, and observations of provider-client consultations provide information on overall facility infrastructure and capacity to provide quality services.

Service Availability Mapping (SAM) collects information on infrastructure, human resources and services at the district and facility level using questionnaires. The SAM team also conducts site visits and records the geographic coordinates of health facilities in order to map their location. SAM was used in Tanzania.

Pia Schneider et al. of Abt Associates used questionnaires, health management information system data, data reported by healthcare providers, firms, insurance companies, and pharmacies to create national health accounts for Rwanda. They then used budget and expenditure information from the Ministry of Finance to fill in any gaps in information left by the questionnaires and other sources.

The Capacity Project aims to improve the integration of FBOs into the national Ministry of Health. In Tanzania, they used GPS to locate health facilities and questionnaires to collect facility data.

The Indonesia record is from a personal communication with a colleague at Universitas Gadja Mada in Indonesia who provided us with Ministry of Health records of facilities nationwide.

Finally, the Wamai paper presented at the International Society for Third Sector Research Sixth International Conference used primary and secondary data and key informant interviews to gather information.
