# Supplementary material for: The Scale of Faith Based Organization Participation in Health Service Delivery in Developing Countries: Systemic Review and Meta-Analysis
Source: PLoS One. 2012 Nov 12;7(11):e48457. doi: 10.1371/journal.pone.0048457 (PMC3495941; doi:10.1371/journal.pone.0048457)
Supplement: Appendix S2 — DHS Regional Breakdown. (DOCX) [file pone.0048457.s002.docx]

**Appendix S2: DHS Regional Breakdown**

Sub-Saharan Africa: Benin 2006, Burkina Faso 2003, Cameroon 2004, Chad 2004, Congo (BR) 2005, Congo DR 2007, Ethiopia 2005, Ghana 2008, Guinea 2005, Kenya 2003, Lesotho 2004, Liberia 2007, Madagascar 2003/04, Malawi 2004, Mali 2006, Mozambique 2003, Namibia 2006/07, Niger 2006, Nigeria 2008, Rwanda 2005, Senegal 2005, Sierra Leone 2008, Swaziland 2006/07, Tanzania 2004/05, Uganda 2006, Zambia 2007, Zimbabwe 2005/06,

Latin America/Caribbean: Bolivia 2003, Colombia 2005, Dominican Republic 2007, Haiti 2005/06, Honduras 2005/06, Peru 2004/08,

Asia/North Africa/Europe: Armenia 2005, Azerbaijan 2006, Egypt 2008, Jordan 2007, Moldova 2005, Morocco 2003/04,

South Asia: Bangladesh 2007, India 2005/06, Nepal 2006, Pakistan 2006/07,

South-East Asia: Cambodia 2005, Indonesia 2007, Philippines 2003
